# Modeling activity patterns of wildlife using time‐series analysis

**DOI:** 10.1002/ece3.2873

**Published:** 2017-03-16

**Authors:** Jindong Zhang, Vanessa Hull, Zhiyun Ouyang, Liang He, Thomas Connor, Hongbo Yang, Jinyan Huang, Shiqiang Zhou, Zejun Zhang, Caiquan Zhou, Hemin Zhang, Jianguo Liu

**Affiliations:** ^1^ Key Laboratory of Southwest China Wildlife Resources ConservationChina West Normal UniversityMinistry of EducationNanchong, Sichuan 637009China; ^2^ Center for Systems Integration and SustainabilityDepartment of Fisheries and WildlifeMichigan State UniversityEast LansingMI 48823USA; ^3^ Department of Wildlife Ecology and ConservationUniversity of FloridaGainesvilleFL 32611USA; ^4^ State Key Laboratory of Urban and Regional EcologyResearch Center for Eco–environmental SciencesChinese Academy of SciencesBeijing 100085China; ^5^ National Meteorological CenterBeijing 100081China; ^6^ Conservation and Research Center for the Giant Panda (CCRCGP)Wolong Nature ReserveSichuan 623006China

**Keywords:** animal behavior, external and internal influences, giant panda (*Ailuropoda melanoleuca*), GPS collar, time‐series analysis

## Abstract

The study of wildlife activity patterns is an effective approach to understanding fundamental ecological and evolutionary processes. However, traditional statistical approaches used to conduct quantitative analysis have thus far had limited success in revealing underlying mechanisms driving activity patterns. Here, we combine wavelet analysis, a type of frequency‐based time‐series analysis, with high‐resolution activity data from accelerometers embedded in GPS collars to explore the effects of internal states (e.g., pregnancy) and external factors (e.g., seasonal dynamics of resources and weather) on activity patterns of the endangered giant panda (*Ailuropoda melanoleuca*). Giant pandas exhibited higher frequency cycles during the winter when resources (e.g., water and forage) were relatively poor, as well as during spring, which includes the giant panda's mating season. During the summer and autumn when resources were abundant, pandas exhibited a regular activity pattern with activity peaks every 24 hr. A pregnant individual showed distinct differences in her activity pattern from other giant pandas for several months following parturition. These results indicate that animals adjust activity cycles to adapt to seasonal variation of the resources and unique physiological periods. Wavelet coherency analysis also verified the synchronization of giant panda activity level with air temperature and solar radiation at the 24‐hr band. Our study also shows that wavelet analysis is an effective tool for analyzing high‐resolution activity pattern data and its relationship to internal and external states, an approach that has the potential to inform wildlife conservation and management across species.

## 1 Introduction

The study of wildlife activity patterns provides insights into evolutionary adaptations, bioenergetics, foraging strategies, and physiological responses to environmental cues (Aschoff, 1966). Activity patterns have been studied across diverse taxa including mammals, birds, amphibians, reptiles, fishes, and insects (Aschoff, 1966). A wealth of information has been learned on topics such as animal competition and temporal niche differentiation, phylogenetic constraints on the time, ecological forces and endogenous factors driving the evolution of activity patterns, and the adjustment of temporal activity in response to human disturbance (Aschoff, 1966; Carter, Shrestha, Karki, Pradhan, & Liu, 2012; Pianka, 1973; Presley, Willig, Castro‐Arellano, & Weaver, 2009; Roll, Dayan, & Kronfeld‐Schor, 2006).

However, current research on wildlife activity patterns suffers from several shortcomings. First, most studies tend to focus on a daily scale driven by environmental variables (e.g., daily light and temperature cycles), with animals categorized as crepuscular, diurnal, or nocturnal (Aschoff, 1966; Lee, Larsen, Flinders, & Eggett, 2010). Limitations of monitoring technologies and analysis techniques have prevented finer scales from being examined. Yet almost all activity patterns appear nonlinear and irregular (Polansky, Wittemyer, Cross, Tambling, & Getz, 2010; Wittemyer, Polansky, Douglas‐Hamilton, & Getz, 2008), as many wildlife species face complex environmental variation and physiological requirements that cannot be accurately described on a daily scale (Alados, Escos, & Emlen, 1996; Escos, Alados, & Emlen, 1995; Kembro, Perillo, Pury, Satterlee, & Marin, 2009; Wittemyer, Polansky, et al., 2008). Second, traditional statistical approaches used in wildlife activity research, such as taking the mean or median value of activity rate or level at a broad temporal resolution and over a short duration, cannot accurately detect the activity characteristics across multitemporal scales, such as hourly, daily, monthly, and yearly (Lee et al., 2010; Polansky et al., 2010; Zhang et al., 2015). Third, traditional approaches have also been limited by the logistical challenge of the inability to control for high autocorrelation inherent in the cyclical activity patterns of wildlife (Loe et al., 2007). General additive models (GAM) and general additive mixed models (GAMM) with smoothing functions have been developed to control for autocorrelation by smoothing temporal variables (e.g., hours or days) (Mandel, Bildstein, Bohrer, & Winkler, 2008; Ryan, Whisson, Holland, & Arnould, 2013; Zhang et al., 2015), but employing smoothing functions often leads to a loss of interpretability (Ryan et al., 2013; Zhang et al., 2015). Furthermore, traditional regression approaches also cannot determine which temporal and spatial scale is optimal for analyzing the relationship between two autocorrelated factors (Ryan et al., 2013; Zhang et al., 2015).

With the advancement of tracking technology, the dynamic motion (e.g., flight, walking, or swimming) of animals can be measured and recorded through animal‐borne data accelerometers (Broell, Taylor, Litvak, Bezanson, & Taggart, 2016; Nakamura, Watanabe, Papastamatiou, Sato, & Meyer, 2011; Sakamoto et al., 2009; Whitney, Pratt, Pratt, & Carrier, 2010). This affords the opportunity to analyze activity patterns using more complex statistical approaches on a finer spatiotemporal scale and also over longer durations of time. One example is time‐series analysis (Sakamoto et al., 2009), a powerful tool used to examine nonlinear and dynamic ecological processes such as population dynamics and ecosystem variation at multiple temporal scales (Bjørnstad & Grenfell, 2001; Cazelles et al., 2008; Rouyer et al., 2008). More recently, time‐series approaches such as Fourier and wavelet analysis have been applied to understand cyclical behavioral properties of individual animals using movement data (Polansky et al., 2010; Wittemyer, Polansky, et al., 2008). These approaches involve analysis of the frequency domain of time‐series data and characterization of dynamics in temporal autocorrelation patterns. Such approaches have helped overcome fundamental challenges related to analyzing highly temporally autocorrelated data and also provided new insights into the periodicity of animal movement and its variation across individuals and seasons. The cyclical switching of movement modes can also reveal strategies that animals use to cope with environmental change (Getz & Saltz, 2008; Nathan et al., 2008; Polansky et al., 2010; Wittemyer, Polansky, et al., 2008). With the increasing availability of high‐resolution activity data, there is now great potential to extend these powerful methods into a new realm of animal behavior to examine animal activity patterns at varied temporal scales. Such an approach would provide new insights into how animals shift their activity patterns on the mean hourly scale over seasons as internal and external conditions change.

Here, we demonstrate the potential of this technique for the first time by applying time‐series analysis methods to analyze activity patterns (e.g., foraging and resting) of five wild giant pandas (*Ailuropoda melanoleuca*) monitored using GPS collars. We sought to identify cyclical activity patterns associated with individual internal states (e.g., pregnancy) and external forcing (e.g., seasonal resource availability and weather). In addition to being a national treasure to China and a flagship species for global environmental conservation, giant pandas are also an ideal study species to test broad ecological hypotheses using telemetry data. Giant panda follow relatively simple behavioral habits, which limits the number of confounding factors that would otherwise hamper the ability to draw inferences. They feed almost exclusively on bamboo, which they digest with low efficiency. Because of this, pandas spend approximate 55% of their time foraging on bamboo, while almost all their remaining time is spent resting (Schaller, Hu, Pan, & Zhu, 1985). Additionally, giant pandas are generally solitary, have relatively stable home ranges, and do not partake in predator–prey interactions (Hull et al., 2015; Schaller et al., 1985; Zhang et al., 2014, 2015). It is also difficult to measure activity patterns by direct observation in this species as the dense forest habitat offers low visibility and pandas avoid humans, making the data all the more valuable (Schaller et al., 1985). In this study, we explored the relationship between the behavior of repetitive cycles between foraging and resting of giant pandas and resource abundance/availability in different seasons (Swets, 1988; Wittemyer, Polansky, et al., 2008). We also tested the relationship between reproductive behaviors and activity cycles of giant pandas, and the relationship between cyclic weather patterns (e.g., air temperature and solar radiation) and panda activity patterns on a daily scale.

## 2 Materials and methods

### 2.1 Data collection

Our study site was located near the Hetaoping Research Base in the northeastern portion of the Wolong Nature Reserve in Sichuan, China (102°52′–103°24′E, 30°45′–31°25′N). Wolong Nature Reserve covers an area of about 2,000 km^2^ and harbors over 100 wild giant pandas(Sichuan Provincial Forestry Department 2015), while our study site encompasses 40–50 km^2^, with an elevation range of approximately 1,800 to 3,400 m. Individual identification through DNA analysis of panda fecal samples collected from May 2012 to November 2013 showed that the Hetaoping area accommodates 22 wild pandas (Huang et al., 2015). There are four forest types—evergreen broad‐leaved forest, deciduous broad‐leaved forest, mixed coniferous and deciduous broad‐leaved forest, and subalpine coniferous forest. Pandas mainly forage on three species of understory bamboo: arrow (*Bashania fangiana*) (above 2,600 m elevation), umbrella (*Fargesia robusta*) (below 2,600 m), and Yushan (*Yushania bravipaniculata*) (1,800–3,400 m) (Li, Zhou, Xiao, Chen, & Tian, 1992).

We fitted GPS collars on five giant pandas (four females and one male) from 2010 to 2011 (Table 1). Veterinarians tranquilized pandas using a compressed‐air gun loaded with 5 mg ketamine/kg of animal weight. GPS collars were then fitted before pandas resumed their normal activities. Data were downloaded periodically via a remote receiver. Licensed veterinarians from the China Conservation and Research Center for the Giant Panda (CCRCGP) assured animal safety. These methods were approved by State Forestry Administration, China, CCRCGP, and The Institutional Animal Care and Use Committee of Michigan State University, USA.

**Table 1:** Summary of GPS‐collared pandas in Wolong Nature Reserve, China, and the duration of overlap between tracking and weather data

Panda	Age	Sex	Duration of tracking	Duration of overlap between tracking and weather data
Mei Mei^a^	Adult	F	04/04/2010–03/29/2012	06/12/2010–09/26/2011
Pan Pan	Adult	F	04/17/2010–11/26/2010	06/12/2010–11/26/2010
Zhong Zhong	Adult	F	03/23/2011–04/03/2012	03/23/2011–09/26/2011
Chuan Chuan	Adult	M	04/06/2011–03/27/2012	04/06/2011–09/26/2011
Long Long	Subadult	F	04/10/2011–10/11/2011	04/10/2011–09/26/2011

a Mei Mei was pregnant around March 2010 and then birthed a cub in August 2010.

We used 12‐channel Lotek GPS_4400 M GPS Collars equipped with dual‐axis activity accelerometers that measured the pandas' activity levels in vertical and horizontal directions (Lotek Engineering Inc., Newmarket, Ontario, Canada). Both directions had a cylinder containing a small sphere. Collars recorded the number of times the spheres hit the cylinder edges in each consecutive 5‐min time interval. Data were recorded on a unitless scale ranging from 0 (no activity) to 255 (highest activity). Vertical and horizontal activity counts were positively correlated (Pearson correlation: all R^2^ > .90, *p *<* *.001). As previous studies showed that accuracy is higher in the vertical than in the horizontal sensor (Coulombe, Massé, & Côté, 2006), we only used activity counts from the vertical sensor in our analysis. We computed the mean hourly activity level to serve as our unit of analysis. We chose this unit of analysis because it was comparable with previous research on giant panda activity patterns that were conducted using mean hourly intervals (Schaller et al., 1985; Zhang et al., 2015). The time span of data collection ranged from 6 months to 2 years per collar (see Table 1). The data were divided into three distinct seasons based on giant panda seasonal foraging strategies (spring—April to June, summer–autumn—July to October, and winter—November to March). For details on the justification of these divisions, see Supporting Information text.

A pilot study that we conducted earlier on one GPS‐collared, captive adult female panda who was also observed using a video monitoring system demonstrated a significant relationship between GPS collar activity counts and panda behavior (Zhang et al., 2015). The activity counts recorded by the GPS collar for inactive behavior (including sleeping, lying, and stationary standing) were significantly lower than activity counts associated with feeding and movement (Zhang et al., 2015). This result supports the validity of the activity count data for representing realized behaviors for this species. For further details on this study, see Zhang et al. (2015).

We monitored air temperature (°C) and solar radiation (W/m^2^) at 5‐min intervals from June 2010 to September 2011 using a meteorological station (HOBO^®^ Microstation with RG3 Rain Gauge, Onset Computer Corporation, Pocasset, MA, USA). This station was located at the Hetaoping Research Base. The distance to the farthest studied panda's activity region was <10 km from the station. Although GPS collars also recorded temperature every 5 min, we did not use this variable for the analysis because previous research has shown that these data are biased by the animals' posture, activity, and pelage type (Maier, Maier, & White, 1996; Schwartz, Podruzny, Cain, & Cherry, 2009).

### 2.2 Time‐series analysis

Wavelet analysis detects the periodic pattern of a time series in both time and frequency domains while handling periodic components, noise, transient dynamics, and intermittent oscillations at a fine resolution (Lau & Weng, 1995; Torrence & Compo, 1998). Because the activity data are typical nonstationary time series, wavelet transform is more suitable than other spectrum analysis techniques (e.g., Fourier transform). Furthermore, wavelet coherence can be used to analyze temporal correlations at different frequencies between two time series (Grinsted, Moore, & Jevrejeva, 2004; Torrence & Compo, 1998). Continuous wavelet transforms (CWT) are one of two types of wavelet transforms and the more appropriate method for extracting features from large signal datasets whose scales are linked to frequencies. CWT is frequently applied to tests on the relationship between two time series on a given temporal scale (Grinsted et al., 2004). We used CWT (Morlet wavelet) to extract common features from wavelike activity signals. We tested for statistical significance against red noise backgrounds using a Monte Carlo method, as theoretical studies have shown that it models a first order autoregressive (AR1) process well (Grinsted et al., 2004). We applied the CWT on each panda's individual time series, with the sampling interval set at ∆*t *= 1 hr. To better examine the effect of physiology, we conducted CWT on the data from one adult female panda (Mei Mei) in the year of her pregnancy (from April 2010 to March 2011) and the next year when she took care of her cub (from April 2011 to March 2012), respectively. We then used wavelet coherence (WTC) to analyze the correlation between panda activity and weather (temperature and solar radiation), and tested for significance of the coherence using Monte Carlo methods. We did not transform the *y*‐axis of CWTs from period (hour) to frequency (cycles/day) like previous studies (Polansky et al., 2010; Wittemyer, Polansky, et al., 2008), because period‐hour more intuitively reflected the temporal band characteristics of panda activity cycles. All computations were run using the cross‐wavelet and wavelet coherence toolbox for the MATLAB software package developed by Grinsted et al. (2004).

## 3 Results

**Figure 1:**
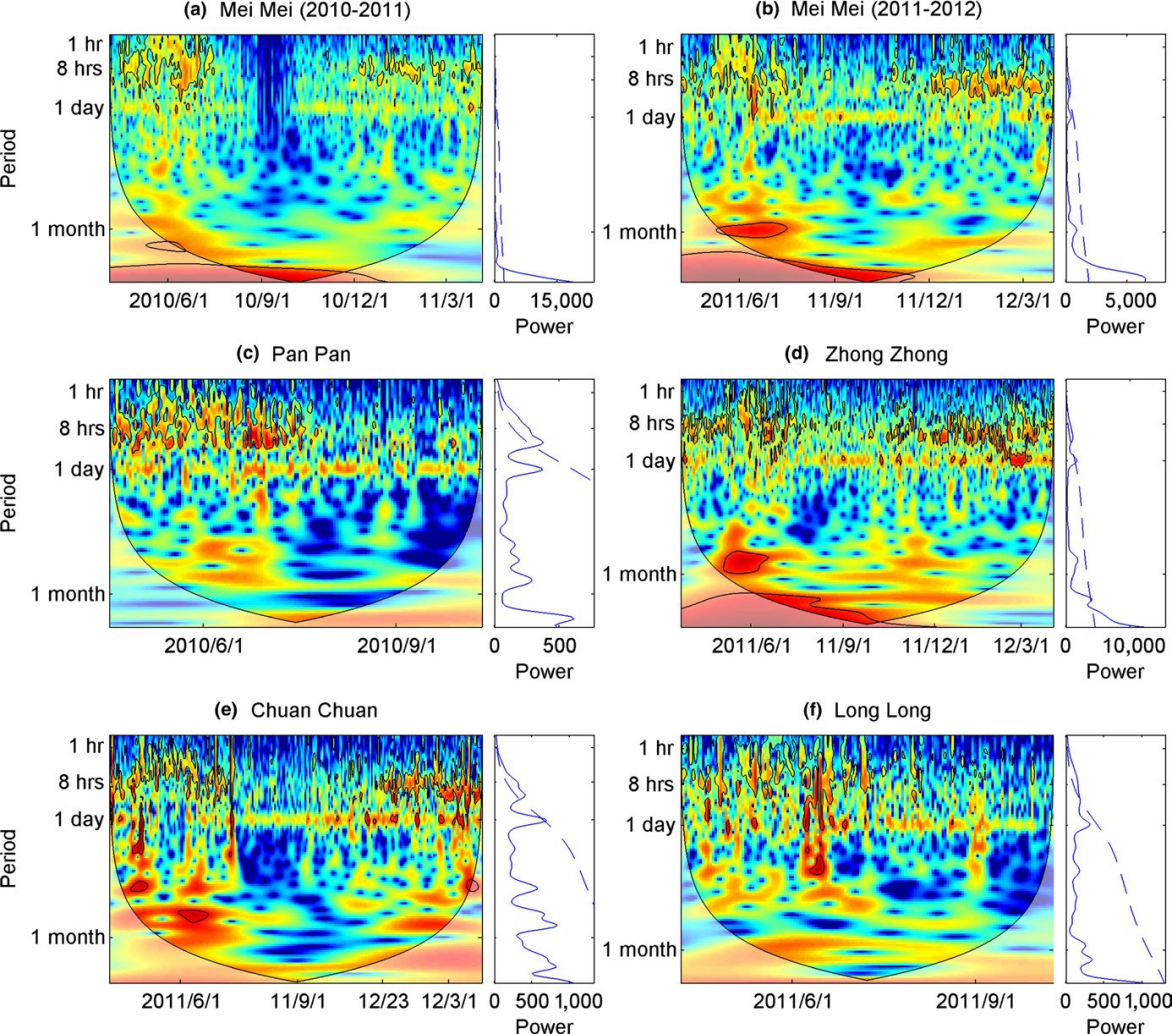
Continuous wavelet power spectrum depicting hourly activity level of five pandas (a: Mei Mei(2010‐2011), b: Mei Mei(2010‐2011), c:Pan Pan, d: Zhong Zhong, e:Chuan Chuan, f: Long Long) monitored using GPS collars (left side of each panel). Black contours designate the 5% significance level against red noise; a large solid line shows the cone of influence outside of which values are impacted by zero padding and should be disregarded. The vertical dashed line represents the estimated date of transition from spring to summer–autumn and winter (spring is from April to June, summer–autumn from July to October, and winter from November to March of the following year). Mei Mei's data were divided into two subsets that were from April 2010 to March 2011, and from April 2011 to March 2012, respectively. The *x*‐axis indicates the date, while the *y*‐axis indicates the time scale in hours. Global wavelet spectrums are shown on the right side of each panel

**Figure 2:**
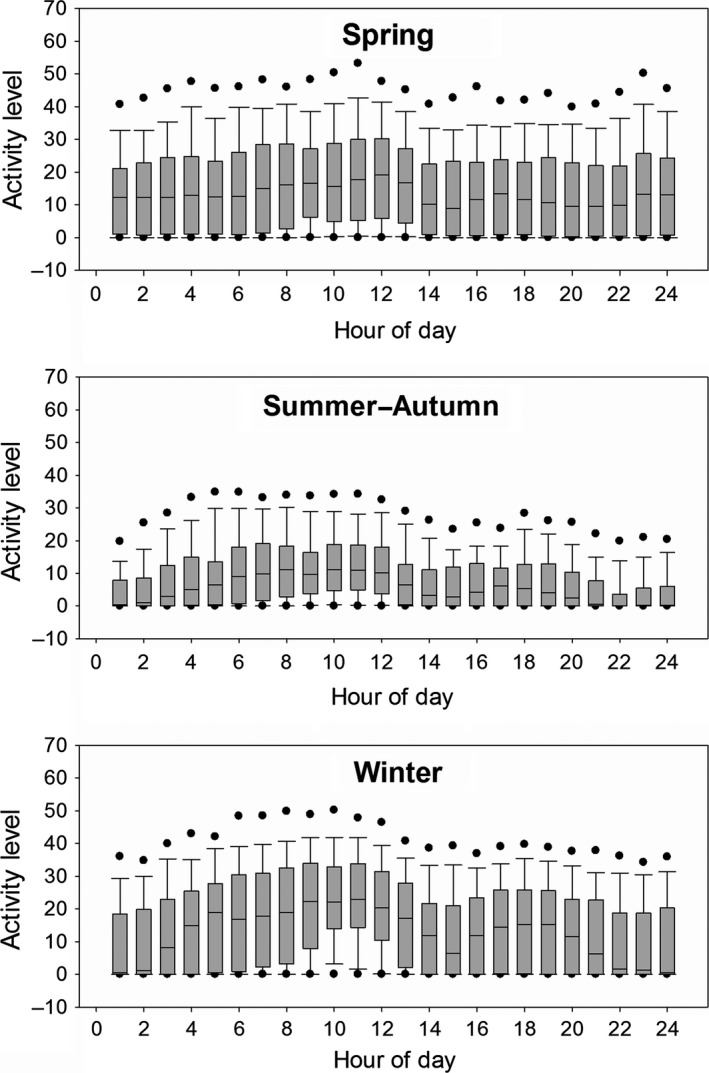
Box plots of average activity level across five giant pandas by time of day during spring, summer–autumn, and winter. The middle line denotes the median value, the box extends from the 25th to the 75th percentiles, and the solid dots denote the values of the 5th and 95th percentiles. We excluded the data of Mei Mei from April 2010 to March 2011, as her activity pattern was abnormal because of pregnancy and parturition in this period

Wavelet analysis showed that pandas had multiple activity cycles in the spring and winter, with two, three, or more activity peaks per day with periodic scales of 12, 8 hr, and <8‐hr time intervals. This pattern is exemplified by a significant peak in the less than half day band which was more than the theoretical mean red noise spectrum (Figure 1). Activity patterns differed during summer–autumn when pandas were diurnal. For all individuals, a common wavelet power spectrum occurred in the 24‐hr band across all seasons (Figure 1). The median value of total active counts also indicated a diurnal activity pattern, with more activity cycles (alternating active peaks and rest periods) during spring–winter than summer–autumn (Figure 2). Across seasons, the dominant activity peak occurred in the daytime reflecting increased activity, with a higher proportion of rest at nighttime (activity count in nighttime vs. daytime: *Z *= −29.039, *p *<* *.001) (Figure 2).

**Figure 3:**
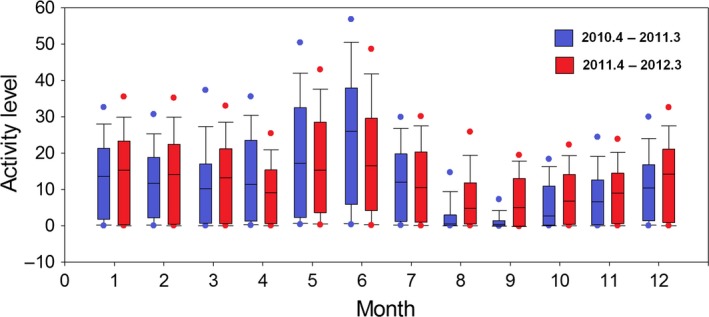
Comparison of Mei Mei's activity level between the year she was pregnant and gave birth (2010–2011) and the following year (2011–2012). She became pregnant in the spring (March or April) of 2010 and gave birth in August of that year. The box plots show average hourly activity level by time of day, where the middle line denotes the median value, the box extends from the 25th to the 75th percentiles, and the solid dots denote the 5th and 95th percentiles

There was distinction between four normal individuals and a particular physiological period of the individual Mei Mei when she was pregnant and until she birthed a cub around August 2010. Mei Mei's activity level was significantly higher during the spring (April–June 2010) she was pregnant compared to the same period the following year (April–June 2011) (*Z *= −7.079, *p *<* *.001) (Figure 3). After June, this disparity in activity level between years began to decrease, and the pattern reversed from August to the following March (lower activity level in the year she was pregnant, Figure 3). Correspondingly, the periodic characteristics of activity patterns also changed during the year. Along with the other studied pandas, Mei Mei had multiple activity cycles before July 2010, but she did not exhibit multiple cyclical features from July to December 2010. The periodogram peak at the 1‐day band was weak in Mei Mei throughout this year, and almost all cyclical characteristics in activity disappeared from the end of August to early October 2010 (Figure 1). After December 2010, her activity periodic cycles began to trend back to normal, similar to other individuals (Figure 1). The periodicity in activity properties of the other four studied pandas was fairly consistent apart from seasonal changes (Figure 1).

**Figure 4:**
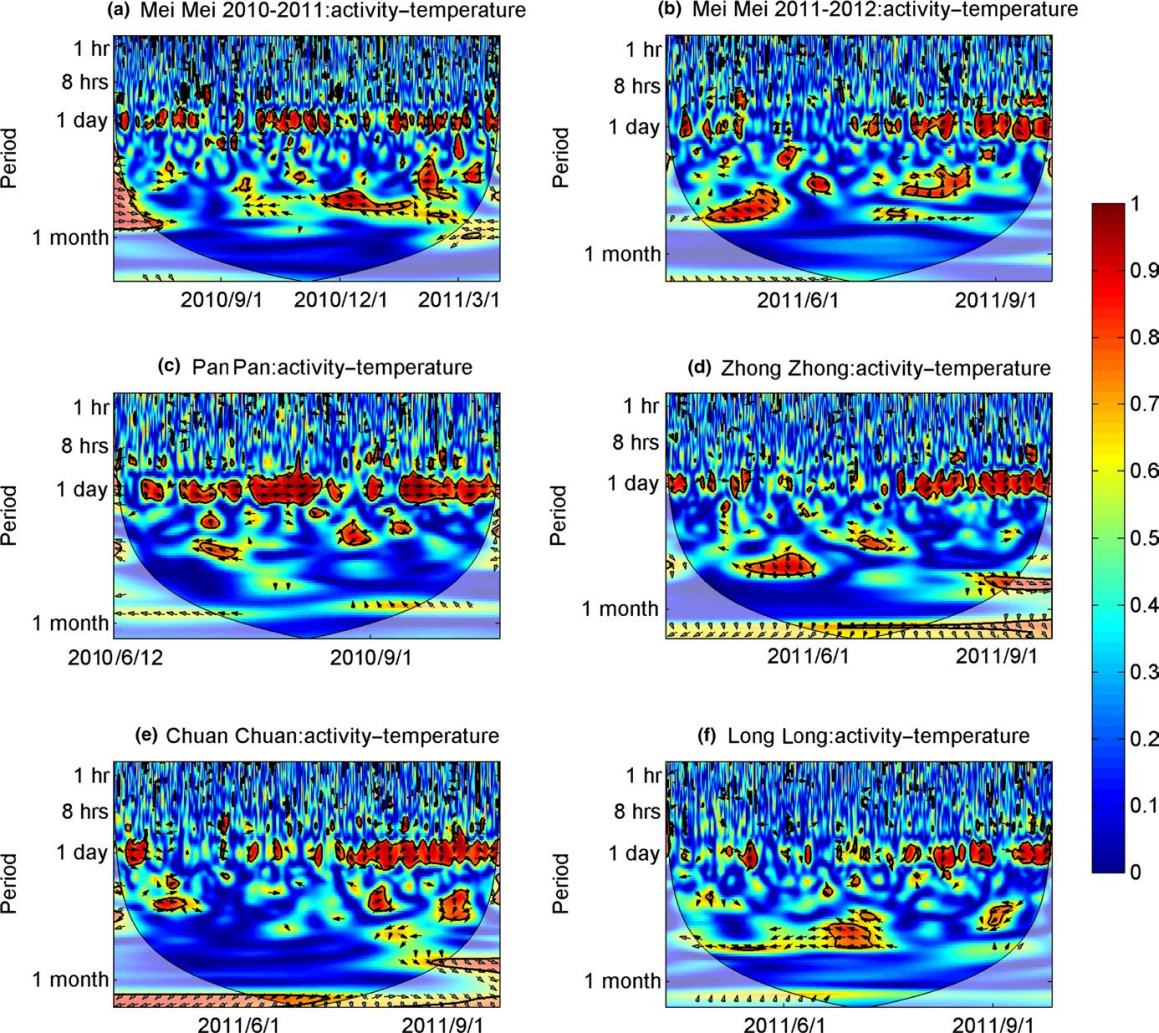
Wavelet coherency between activity level of five giant pandas (a: Mei Mei(2010‐2011), b: Mei Mei(2010‐2011), c:Pan Pan, d: Zhong Zhong, e:Chuan Chuan, f: Long Long) monitored using GPS collars and temperature. The 5% significance level against red noise is shown as a black contour, the color bar indicates strength of correlation, and the direction of the arrow indicates phase information or the type of correlation (right directed—”in phase or positive”; left directed—”antiphase or negative”; down—X leading Y by 90°; up—Y leading X by 90°). The *x*‐axis indicates the date, while the *y*‐axis indicates the timescale in hours. Mei Mei's data were divided into two subsets which were from April 2010 to March 2011, and from April 2011 to March 2012, respectively

**Figure 5:**
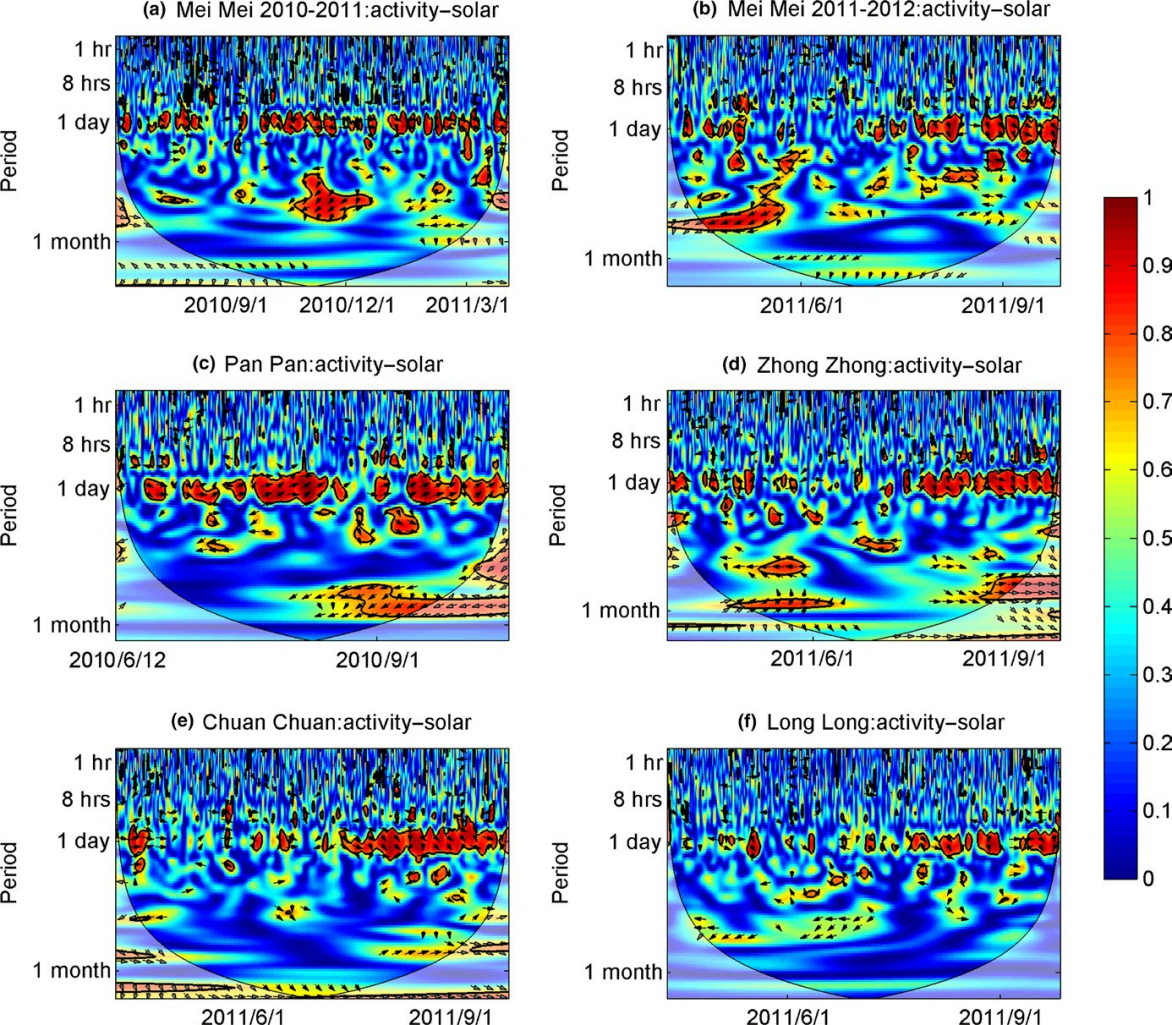
Wavelet coherency between activity level of five giant pandas (a: Mei Mei (2010‐2011), b: Mei Mei(2010‐2011), c:Pan Pan, d: Zhong Zhong, e: Chuan Chuan, f: Long Long) monitored using GPS collars and solar radiation. The 5% significance level against red noise is shown as a black contour, the color bar indicates the strength of correlation, and the direction of the arrow indicates phase information or the type of correlation (right directed—”in‐phase or positive”; left directed—”antiphase or negative,”; down—X leading Y by 90°; up—”Y leading X by 90°). The *x*‐axis indicates the date, while the *y*‐axis indicates the timescale in hours. Mei Mei's data were divided into two subsets which were from April 2010 to March 2011, and from April 2011 to March 2012, respectively

There were in‐phase (positive) coherencies at the 24‐hr band between activity level and air temperature across each season (Figure 4). The average coherency was 0.61 ± 0.07 and ranged from 0.56 to 0.73, with an average time lag of 15.03 ± 7.18 hr (Table 2). The relationship between solar radiation and panda activity level was similar and also exhibited in‐phase coherencies (Figure 5). The average coherency in this relationship was 0.64 ± 0.04 and ranged from 0.62 to 0.71, with an average time lag of 17.73 ± 7.23 hr (Table 2). The coherency within a single individual (Mei Mei) was similar in different years, and the time lag was similar as well (Table 1). But responses to weather varied across pandas.

**Table 2:** Relationship between activity level of GPS‐collared giant pandas and weather (temperature and solar radiation). Mean coherence and time lag were measured on a daily scale (24 hr)

Panda	Temperature activity		Solar activity	
	Mean coherence	Time lag (hours)	Mean coherence	Time lag (hours)
Mei Mei (2010–2011)	0.56	18.03	0.64	21.19
Mei Mei (2011–2012)	0.56	18.00	0.64	21.20
Pan Pan	0.73	0.43	0.71	2.66
Zhong Zhong	0.61	17.87	0.62	19.81
Chuan Chuan	0.64	18.81	0.64	20.05
Long Long	0.58	17.09	0.58	19.01
Mean ± *SD*	0.61 ± 0.07	15.03 ± 7.18	0.64 ± 0.04	17.32 ± 7.23

Results are extracted from the red areas corresponding to the daily scale in the wavelet coherence analysis of Figures 4 and 5.

A positive time lag means that the response of the first variable is before, by x number of hours, the response of the second variable.

## 4 Discussion

In this study, we applied continuous wavelet transforms (CWT) and wavelet coherency analysis to identify the activity patterns of wildlife. While there have been successful applications of this method to understand similar processes using animal movement data (Polansky et al., 2010; Wittemyer, Polansky, et al., 2008), extending the approach into a new dimension of animal activity data opened up new avenues of inquiry (Sakamoto et al., 2009). Time‐series analysis of activity patterns may more accurately reflect the mechanisms of energy cost and maintenance than traditional methods in movement ecology. Both processes allow for inference to be made on an animal's survival status, physiological state, and adaptive response to environmental stimuli. But activity pattern data overcome the large error in turning angles (Zollner & Lima, 1999) and the gross underestimation of movement distances from successive GPS locations (Mandel et al., 2008).

The autocorrelation properties of animal activity patterns are interesting to study because they may reflect differences in physiological states of animals and their responses to environmental factors in new ways (MacIntosh, Alados, & Huffman, 2011; MacIntosh, Pelletier, Chiaradia, Kato, & Ropert‐Coudert, 2013). For example, some studies have shown that physiological stressors (e.g., clinically impaired health, reproductive activities) or other challenges (e.g., low dominance status) are associated with less stochasticity, that is, increasing periodicity or stereotypy (Alados & Weber, 1999; Alados et al., 1996; Motohashi, Miyazaki, & Takano, 1993; Rutherford, Haskell, Glasbey, & Lawrence, 2006; Seuront & Cribb, 2011). In contrast, individuals show increased complexity of behavioral patterns when they explore resources in novel environments, which in turn may increase foraging success rates (Alados et al., 1996; Escos et al., 1995; Kembro et al., 2009; MacIntosh et al., 2011; Shimada, Minesaki, & Hara, 1995). Most previous studies mainly use fractal analysis on binary datasets (e.g., stationary vs. nonstationary data) to analyze autocorrelation of animals' activity patterns associating with endogenous and exogenous factors (MacIntosh et al., 2011, 2013). However, such approaches cannot assess variation in autocorrelation properties along continuous, high‐resolution time scales that may be more sensitive for picking up nuances in responses to internal and external stimuli (MacIntosh et al., 2011, 2013). Our application of wavelet analysis provided a more robust statistical approach to analyzing autocorrelation of activity pattern data on a continuous scale across context‐specific physiological and environmental factors (MacIntosh et al., 2013; Wittemyer, Polansky, et al., 2008).

Our study also revealed new insights into activity patterns of the endangered giant panda. Previous studies have been carried out on activity patterns in pandas, but they have been limited to traditional approaches (Schaller et al., 1985; Zhang et al., 2015). Our findings expand upon this research by demonstrating variation not only in panda activity levels but also pandas' frequency‐based cyclical activity modes. For example, earlier studies showed that giant pandas exhibit an activity valley during summer–autumn, likely relating to the high quality food and easy access to water (Nie, Speakman, et al., 2015; Schaller et al., 1985; Zhang et al., 2015). This season was also distinct in our study in that it was the only season in which we found a diurnal activity cycle. Pandas perhaps displayed more frequent cycles during spring due to the demands of mating activities and in winter due to declines in food and water quality and/or availability relative to summer–autumn. During spring, pandas move from high to low elevation areas to forage on umbrella bamboo shoots which contain high nutrition (Table S1) (Nie, Zhang, et al., 2015), and need to make long‐distance movements (more energy expenditure) to pursue new bamboo shoots (Schaller et al., 1985; Zhang et al., 2015). Moreover, spring is the mating season for pandas—male pandas roam an extensive range to encounter females and fight for mating rights. During winter, pandas need to spend more time foraging for food and water resources. In our field observation and previous studies, pandas visited a few permanent water sites more often in winter as temporary pool sites disappeared and most streams froze (Schaller et al., 1985; Zhang et al., 2014). The food available during this season (stems and old shoots) is also less nutritious than other times of year. In addition, a recent study showed that pandas' net energy assimilation (NEA) and associated metabolic rate was negatively related to daily shade temperature, another potential reason for the lower activity and less frequent activity cycling in summer and autumn than other seasons (Nie, Speakman, et al., 2015). Similar behavioral strategies were also found in movement studies on other species—energy budgeting associated with diet and water resources are major explaining factors of shifts in animal behavioral (e.g., forage or movement) cycles across seasons (Wittemyer, Polansky, et al., 2008; Zollner & Lima, 1999).

Reproduction is a cyclic behavior influenced by predictable phenomena such as circadian light and temperature (Prendergast, Nelson, & Zucker, 2002). However, the change in cyclical behavior patterns relating to breeding activity has rarely been investigated due to the lack of long‐term empirical datasets (Wittemyer, Polansky, et al., 2008). A pregnant female (Mei Mei) in this study provided a rare opportunity to test hypotheses concerning cyclical activity patterns in the breeding period. The near disappearance of the diurnal band in her data during summer–autumn 2010 suggests that mother pandas follow abnormal activity patterns for around 5 months after parturition (Figure 1). This result is consistent with previous field observations in the Qinling mountains, where cubs start walking around in the forest after the age of 5 months and mothers begin to recover to normal activity levels(Pan et al., 2014). It makes sense that Mei Mei's activity level was significantly higher during the spring she was pregnant than the same period in the next year, as she had to build up a storage of energy during the pregnancy (Figure 3). Pregnant females need to forage more bamboo shoots that emerge during the spring season to satisfy breeding requirements (Zhang et al., 2015). Shoots have high 6‐methoxy‐2‐benzoxazolinone (6‐MBOA) content, which is advantageous for embryonic development and the survival of offspring (Nelson, 1991; Rosenfeld & Shelby, 2004).

Even considering the multiple‐cycles/day activity pattern of pandas during spring and winter, pandas still displayed a common diurnal cycle across seasons (the only exception being Mei Mei in her first month after parturition) (Figure 1). Although we cannot infer causality from our results, the coherency at the 24‐hr band scale between activity level and temperature/solar radiation across all pandas throughout the study period (Figures 4, 5 and Table 2) suggests that there may be a relationship between weather cycles and panda activity. This hypothesis is supported by previous analysis of this same dataset using GAMM did not detect a significant effect of temperature on panda activity, but did find a significantly positive correction between solar radiation and activity level for four (out of 5) of the studied pandas (Zhang et al., 2015).

The giant panda's simple behavioral habits make the species an ideal study subject for wavelet analysis, but this is also a constraint for expanding the approach to other wildlife that may have more complex behavior patterns that encompass a greater number of behavior categories. Further research is needed to better classify ethograms of wildlife with larger numbers of behavioral categories, such as birds and fish in a way that would allow for integration with wavelet analysis (Broell et al., 2016; Nakamura et al., 2011; Sakamoto et al., 2009). And more empirical studies with high‐resolution and long‐term data also are still required to fully explore relationships between cycles in animal behavior and concurrent cycles in environmental characteristics (Wittemyer, Polansky, et al., 2008). We also suggest that research into the relationship between physiological strategies and phase differences between rhythmic activity modes (e.g., daily and seasonal activity, breeding cycles) should be extended to more empirical studies. Future studies could also integrate activity data together with movement data to draw stronger conclusions about changes in animal behavior under varying internal and external conditions. Additional research should consider the influence of periodic human activities (e.g., seasonal resource collection) on animals' behavior, as the competition over resources and space between humans and wildlife is increasingly intense in today's human‐dominated world (Wittemyer, Elsen, Bean, Burton, & Brashares, 2008; Woodroffe, Thirgood, & Rabinowitz, 2005).

## Conflict of interest

The authors declare no conflict of interest.

## Author contributions

J.Z., V.H., Y.O., and J.L. designed research; J.Z., V.H., J.H., S.Z., Z.Z., C.Z., and H.Z. performed research; J.Z., L.H. and H.Y., analyzed data; J.Z., V.H., and T.C., H.Y., and J.L. wrote the manuscript.
