# Supplementary material for: Modeling activity patterns of wildlife using time‐series analysis
Source: Ecol Evol. 2017 Mar 16;7(8):2575–84. doi: 10.1002/ece3.2873 (PMC5395454; doi:10.1002/ece3.2873)
Supplement: Supplementary file 1 [file ECE3-7-2575-s001.docx]

**Supporting Information (SI):**

**Modeling activity patterns of wildlife using time series analysis**

**SI Text**

**Ecological seasons for the giant panda in Wolong Nature Reserve**

The giant panda is a dietary specialist that consumes mainly bamboo. The giant panda alternates between different bamboo species and plant parts according to the time of year, resulting in three distinct ecological seasons in Wolong ([1](#_ENREF_1)). In the spring, from April to June, pandas mainly forage on the shoots of umbrella bamboo and the stems of arrow and Yushan bamboo. Umbrella bamboo shoots are preferred because they contain abundant water and a high proportion of protein, and have good palatability ([2](#_ENREF_2)). Previous studies have shown that the shoots have a high [6-methoxy-2-benzoxazolinone](http://www.surechem.org/index.php?Action=chemical_info&name=6-methoxy-2-benzoxazolinone) (6-MBOA) content which can facilitate embryonic development and improve offspring survival ([3](#_ENREF_3), [4](#_ENREF_4)). Integrating giant panda locations from GPS collars with the distribution range of umbrella bamboo, we verified that giant pandas forage on shoots for around 1-2 months from the end of April or the beginning of May to the beginning of July (Table S2). Spring is also the mating season of giant pandas, and usually lasts from late March to the middle of May ([1](#_ENREF_1)). During this period, both males and females need to forage more food and gain more energy to meet the physiological requirements of reproduction. In summer-autumn, from July to October, pandas primarily consume the leaves and twigs of arrow and Yushan bamboo, new leaves containing the highest concentration of digestible energy and protein of the whole year. During this time, pandas can also obtain water from wet bamboo ([1](#_ENREF_1)), as well as gullies and temporary water pools distributed throughout the habitat. Winter lasts from November to March of the following year. During this time most bamboo leaves are sere and fallen, and giant pandas shift to stems and old shoots (less than one year old) of arrow and Yushan bamboo, containing less nutrition than other bamboo parts ([2](#_ENREF_2)). Temporary water pools disappear and almost all streams freeze, forcing giant pandas to visit a smaller number of permanent water resources.

1. Schaller GB, Hu J, Pan W, & Zhu J (1985) *The giant pandas of Wolong* (University of Chicago Press Chicago, Illinois, USA).

2. Hu J (2001) The studies of the giant panda. *Shanghai Public House of Science and Technology, Shanghai, China (In Chinese)*.

3. Nelson RJ (1991) Maternal diet influences reproductive development in male prairie vole offspring. *Physiology & behavior* 50(5):1063-1066.

4. Rosenfeld M & Shelby N (2004) Methods for augmenting immune defenses contemplating the administration of phenolic and indoleamine-like compounds for use in animals ans humans. (Google Patents).

**Table S1. Spring elevational migration patterns of GPS-collared giant pandas. Start dates for the move from low elevation (umbrella bamboo) to high elevation (arrow bamboo) are shown, as are start dates for the subsequent move back to low elevation. Elevational differences between the start date and one day after are also shown.**

| Panda | Start date of move to low elevation | Elevation change in one day (m) | Start date of move to high elevation | Elevation change in one day (m) |
| --- | --- | --- | --- | --- |
| Mei Mei(2010) | 8 May | 2712/2491 | 3 Jul | 2653/2953 |
| Mei Mei(2011) | 6 May | 2743/2488 | 19 Jun | 2599/2761 |
| Pan Pan(2010) | 20 Apr | 2742/2494 | 12 Jul | 2686/2799 |
| Zhong Zhong(2011) | 6 May | 2741/2654 | 8 Jul | 2660/2892 |
| Chuan Chuan(2011) | 8 May | 2743/2276 | 16 Jun | 2429/2733 |
| Long Long(2011) | 9 May | 2755/2455 | 23 Jun | 2539/2896 |
